# What is polypharmacy? A systematic review of definitions

**DOI:** 10.1186/s12877-017-0621-2

**Published:** 2017-10-10

**Authors:** Nashwa Masnoon, Sepehr Shakib, Lisa Kalisch-Ellett, Gillian E. Caughey

**Affiliations:** 1 Quality Use of Medicines and Pharmacy Research Centre, School of Pharmacy and Medical Sciences, University of South Australia, Frome Road, Adelaide, South Australia Australia; 2 Department of Pharmacy, Royal Adelaide Hospital, North Terrace, Adelaide, South Australia Australia; 3 Department of Clinical Pharmacology, Royal Adelaide Hospital, North Terrace, Adelaide, South Australia Australia; 4 Discipline of Pharmacology, School of Medicine, University of Adelaide, North Terrace, Adelaide, South Australia Australia

**Keywords:** Polypharmacy, Multimorbidity, Comorbidity, Inappropriate prescribing, Aged, Systematic review

## Abstract

**Background:**

Multimorbidity and the associated use of multiple medicines (polypharmacy), is common in the older population. Despite this, there is no consensus definition for polypharmacy. A systematic review was conducted to identify and summarise polypharmacy definitions in existing literature.

**Methods:**

The reporting of this systematic review conforms to the Preferred Reporting Items for Systematic reviews and Meta-Analyses (PRISMA) checklist. MEDLINE (Ovid), EMBASE and Cochrane were systematically searched, as well as grey literature, to identify articles which defined the term polypharmacy (without any limits on the types of definitions) and were in English, published between 1st January 2000 and 30th May 2016. Definitions were categorised as i. numerical only (using the number of medications to define polypharmacy), ii. numerical with an associated duration of therapy or healthcare setting (such as during hospital stay) or iii. Descriptive (using a brief description to define polypharmacy).

**Results:**

A total of 1156 articles were identified and 110 articles met the inclusion criteria. Articles not only defined polypharmacy but associated terms such as minor and major polypharmacy. As a result, a total of 138 definitions of polypharmacy and associated terms were obtained. There were 111 numerical only definitions (80.4% of all definitions), 15 numerical definitions which incorporated a duration of therapy or healthcare setting (10.9%) and 12 descriptive definitions (8.7%). The most commonly reported definition of polypharmacy was the numerical definition of five or more medications daily (*n* = 51, 46.4% of articles), with definitions ranging from two or more to 11 or more medicines. Only 6.4% of articles classified the distinction between appropriate and inappropriate polypharmacy, using descriptive definitions to make this distinction.

**Conclusions:**

Polypharmacy definitions were variable. Numerical definitions of polypharmacy did not account for specific comorbidities present and make it difficult to assess safety and appropriateness of therapy in the clinical setting.

## Background

Multimorbidity, commonly defined as the co-existence of two or more chronic health conditions, is common in the older population [1]. The presence of multiple chronic conditions increases the complexity of therapeutic management for both health professionals and patients, and impacts negatively on health outcomes. Multimorbidity is associated with decreased quality of life, self-rated health, mobility and functional ability as well as increases in hospitalisations, physiological distress, use of health care resources, mortality and costs [2–4]. Globally, the health burden of multimorbidity is expected to rise significantly as a result of the growing number of older people and increasing numbers of people living with multimorbidity [5].

The use of multiple medicines, commonly referred to as polypharmacy is common in the older population with multimorbidity, as one or more medicines may be used to treat each condition. Polypharmacy is associated with adverse outcomes including mortality, falls, adverse drug reactions, increased length of stay in hospital and readmission to hospital soon after discharge [6–8]. The risk of adverse effects and harm increases with increasing numbers of medications [9]. Harm can result due to a multitude of factors including drug-drug interactions and drug-disease interactions. Older patients are at even greater risk of adverse effects due to decreased renal and hepatic function, lower lean body mass, reduced hearing, vision, cognition and mobility [10].

While in many instances the use of multiple medicines or polypharmacy may be clinically appropriate, it is important to identify patients with inappropriate polypharmacy that may place patients at increased risk of adverse events and poor health outcomes. Studies have suggested a shift towards adopting the term ‘appropriate polypharmacy’ in order to differentiate between the prescribing of ‘many’ and ‘too many’ drugs instead of a simple numerical count of medications, which is of limited value in practice [11, 12]. In order to make this distinction between appropriate and inappropriate polypharmacy, the term polypharmacy needs to be clearly defined. We therefore conducted a systematic review to explore the definitions of polypharmacy in existing literature. We additionally aimed to explore whether articles differentiated between appropriate and inappropriate polypharmacy and how this distinction was made.

## Methods

### Data sources and search strategy

The reporting of this systematic review conforms to the PRISMA (Preferred Reporting Items for Systematic reviews and Meta-Analyses) checklist.

MEDLINE (Ovid), EMBASE and Cochrane databases were searched between 1st January 2000 and 30th May 2016.

The following search terms (Medical Subject Headings or MESH and keywords) were used in EMBASE and MEDLINE (Ovid):

polypharmacy/ (MESH) OR multiple medication* OR multiple medicine* OR multiple drug* (key words) OR many medication* OR many medicine* OR many drug* (key words) (for all articles referring to polypharmacy) AND.

defin* (key word) or explan* (keyword) (for all articles defining or explaining polypharmacy).

For the review of the Cochrane database, the term “polypharmacy” was searched.

The search was limited to primary research articles which defined the term polypharmacy in any shape or form, conducted in humans and published in English between the years 2000 and 2016. Articles were considered if the abstracts were available in English and were published or in press. Reference lists of relevant articles and grey literature were screened to identify other relevant articles. The search strategy was developed in consultation with a librarian specialising in health databases, with a pre-determined protocol developed collaboratively with the authors for methods to search and select relevant articles.

### Study selection and data extraction

Articles that met the inclusion criteria and provided a definition of polypharmacy were included. One author (NM) conducted the initial database search and primary screening of article titles and abstracts and articles were categorised as: relevant, irrelevant or unsure. Three reviewers (NM, SS, GC) discussed the appropriateness of inclusion of each article classed as relevant or unsure. Once all relevant articles were identified, one author (NM) reviewed full texts of all identified articles and extracted the data. A pre-defined data extraction template was developed by all authors and then applied to ensure consistent data extraction from each of the identified studies. Data items extracted included the definitions of polypharmacy and associated terms such as minor, moderate and excessive polypharmacy and whether studies distinguished between appropriate and inappropriate polypharmacy and if so, how this distinction was made or defined. The definitions of polypharmacy and associated terms were categorised as: i. numerical only (using the number of medications to define polypharmacy), ii. numerical for a given duration of therapy or healthcare setting for e.g. during hospital stay or iii. Descriptive (using a brief description to define polypharmacy). Once the primary data extraction was complete all authors reviewed the content analysis for each of the extracted studies, with data further categorised and summarised in tables.

## Results

**Fig. 1:**
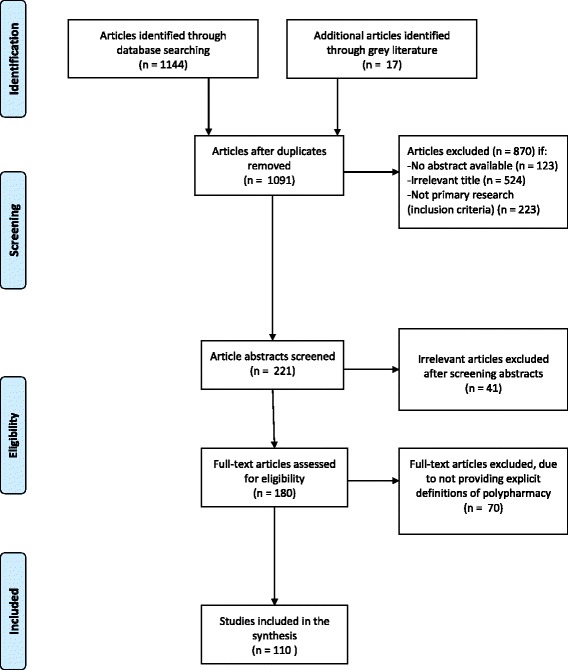
Study selection flowchart according to PRISMA checklist

A total of 1156 articles were identified and 110 articles met the full inclusion criteria for this systematic review [10–119]. Fig. 1 shows a flowchart of study selection according to the PRISMA checklist.

Studies not only defined polypharmacy but also used associated terms to define the level of polypharmacy; including minor (8 studies, 7.3%), moderate (1 study, 0.9%), major (12 studies, 10.9%), hyper (2 studies, 1.8%), excessive (10 studies, 9.1%), severe (1 study, 0.9%), appropriate (1 study, 0.9%), rational polypharmacy and indiscriminate prescribing (1 study, 0.9%), persistent (1 study, 0.9%), chronic (1 study, 0.9%), and pseudopolypharmacy (1 study, 0.9%). As a result, a total of 138 definitions of polypharmacy and associated terms were obtained. There were 111 numerical only definitions (80.4% of all definitions), 15 numerical definitions which incorporated a duration of therapy or healthcare setting (10.9%) and 12 descriptive definitions (8.7%). Table 1 presents a breakdown of the number of definitions for each term.

**Table 1:** Breakdown of polypharmacy definitions according to the category of definition

Term	Numerical only	Numerical in a given duration of time or setting	Descriptive	Total number of definitions
Polypharmacy	81	9	9	99
Minor Polypharmacy	8	0	0	8
Moderate polypharmacy	1	0	0	1
Major polypharmacy	11	1	0	12
Hyperpolypharmacy	1	1	0	2
Excessive polypharmacy	8	2	0	10
Severe polypharmacy	1	0	0	1
Persistent polypharmacy	0	1	0	1
Chronic polypharmacy	0	1	0	1
Appropriate polypharmacy	0	0	1	1
Rational polypharmacy and indiscriminate prescribing	0	0	1	1
Pseudopolypharmacy	0	0	1	1
Total number of definitions according to category of definition	111	15	12	138

Out of the 110 identified articles, 81 (73.6%) included only a numerical definition of polypharmacy (i.e. did not specify duration of therapy or healthcare setting). Nine articles (8.2%) included numerical definitions of polypharmacy for a given duration of time or healthcare setting and nine articles (8.2%) included descriptive definitions of polypharmacy. Four articles included two categories of polypharmacy definitions: two articles (1.8%) included both numerical only definitions and numerical definitions of polypharmacy for a duration of time or healthcare setting and two articles (1.8%) included both numerical only and descriptive definitions of polypharmacy.

### Numerical only definitions of polypharmacy in existing literature

Table 2 shows the various numerical only categorisations of polypharmacy and associated terms and the number of studies using these definitions.

**Table 2:** Various numerical only definitions of polypharmacy and associated terms in existing literature

Term	Number of medications	Number of studies	References
Polypharmacy	≥ 2	1	[13]
	2 to 9	1	[14]
	≥ 3	1	[15]
	3 to 6	1	[16]
	≥ 4	6	[17–22]
	≥ 4 or ≥ 5	1	[23]
	≥ 5	51	[11, 24–73]
	≥ 6	10	[10, 74–82]
	≥ 7	2	[83, 84]
	5 to 9	3	[85–87]
	≥ 9	1	[88]
	≥ 10	1	[89]
	≥ 11	1	[90]
	number of drug classes	1	[91]
Minor Polypharmacy	2 to 4	6	[92–97]
	2 to 3	1	[98]
	0 to 4	1	[99]
Moderate polypharmacy	4 to 5	1	[98]
Major polypharmacy	≥ 5	6	[92–95, 97, 100]
	≥ 6	3	[96, 98, 101]
	5 to 9	1	[99]
	≥ 11	1	[74]
Hyperpolypharmacy	≥ 10	1	[102]
Excessive polypharmacy	≥ 10	7	[30, 58, 65, 70, 85–87]
	≥ 21	1	[74]
Severe polypharmacy	≥ 10	1	[99]

There was a wide range of variability in the definitions of polypharmacy as well as associated terms such as minor, moderate and major polypharmacy. The most commonly used term was polypharmacy, but there was variation with regard to the actual definition of polypharmacy, which ranged from two or more medications to 11 or more medications [13, 90]. The most commonly used definition for polypharmacy was five or more medications daily, with 46.4% (*n* = 51) of studies using this definition [11, 24–73]. The second most common definition for polypharmacy was six or more medications, with ten studies using this definition [10, 74–82]. Only one study defined polypharmacy as the number of drug classes used by a patient [91].

### Numerical definitions of polypharmacy incorporating a duration of therapy or healthcare setting

Eleven studies (10.0% of all studies) used numerical definitions of polypharmacy which incorporated a duration of therapy in the definition and four studies (3.6%) used definitions of polypharmacy which incorporated a healthcare setting (Table 3). The definitions of polypharmacy involving a duration of therapy, ranged from use of two or more medications for more than 240 days (‘long term use’) to five to nine medications used for 90 days or more [101, 108]. Polypharmacy definitions incorporating a healthcare setting included the use of five or more medications at hospital discharge, and the use of 10 or more medications during hospital stay [106, 110].

**Table 3:** Numerical definitions of polypharmacy and associated terms by duration of therapy/ healthcare setting

Term	Number of medications	Number of studies	References
Polypharmacy	≥ 2 for > 240 days (long term)	1	[101]
	≥ 5 medications in the same month	1	[103]
	> 5 medications for ≥ 90 days	1	[104]
	≥ 5 medications in the same quarter of a year	1	[105]
	≥ 5 medicines at hospital discharge	1	[106]
	5 to 9 medicines on the day of maximum number of prescriptions of the study year (on the day of the study year when the number of medications prescribed was highest)	1	[107]
	5 to 9 medications for ≥ 90 days	1	[108]
	5 to 9 medicines during hospital stay	1	[109]
	≥ 10 medicines during hospital stay	1	[110]
Major polypharmacy	≥ 10 on the day of maximum number of prescriptions of the study year (on the day of the study year when the number of medications prescribed was highest)	1	[107]
Hyperpolypharmacy	≥ 10 medications for ≥90 days	1	[108]
Excessive polypharmacy	≥ 10 medications in the same quarter of a year	1	[105]
	≥ 10 medications during hospital stay	1	[109]
Persistent polypharmacy	≥ 5 medications for 181 days	1	[52]
Chronic polypharmacy	≥ 5 medications in 1 month for 6 months (consecutive or not) in a year	1	[111]

### Descriptive definitions of polypharmacy

Twelve studies used descriptive definitions of polypharmacy (Table 4). Some studies used different wording but conveyed the same definition of polypharmacy. For example, the definitions “Co-prescribing multiple medications” [113] and “Simultaneous and long term use of different drugs by the same individual” [77] describe polypharmacy as the use of multiple medications concurrently. Other studies alluded to a different issue of medications being appropriate or inappropriate for a given patient [10, 79, 114–118].

**Table 4:** Descriptive definitions of polypharmacy and associated terms

Term	Definition	Number of studies	References
Polypharmacy	Patients visiting multiple pharmacies to obtain medications	1	[112]
	Coprescribing multiple medications	1	[113]
	Simultaneous and long term use of different drugs by the same individual	1	[77]
	Polypharmacy definition ranges from the use of a large number of medications, to the use of potentially inappropriate medications, medication underuse and medication duplication	1	[114]
	Potentially inappropriate medications	2	[10, 79]
	Use of multiple medications concurrently and the use of additional medications to correct adverse effects	1	[115]
	Use of medications which are not clinically indicated	1	[116]
	More drugs being prescribed or taken than are clinically appropriate in the context of a patient’s comorbidities	1	[12]
Appropriate polypharmacy	Optimisation of medications for patients with complex and/or multiple conditions where medicine usage agrees with best evidence	1	[117]
Rational polypharmacy and indiscriminate prescribing	Rational polypharmacy recognizes legitimate prescribing and indiscriminate prescribing suggests inappropriate prescribing (the terms “legitimate prescribing” and “inappropriate prescribing” were not explained)	1	[118]
Pseudopolypharmacy	Patients being recorded as taking more medications than they are actually taking	1	[119]

### Appropriate and inappropriate polypharmacy

Only seven studies (6.4% of all studies) defined appropriate or rational polypharmacy, or recognised the distinction between appropriate and inappropriate medications [10, 79, 114–118]. These studies either defined polypharmacy using a brief description only (*n* = 3) [79, 115, 117] or used a brief description and polypharmacy tools such as the Beers criteria and the Medication Appropriateness Index (MAI) (*n* = 4 studies) [10, 114, 116, 118]. An example of a polypharmacy definition which recognised the use of appropriate and inappropriate medications is “polypharmacy ranges from the use of a large number of medications, to the use of potentially inappropriate medications, medication underuse and duplication” and “potentially inappropriate medications” [114]. Out of the two studies defining polypharmacy as “potentially inappropriate medications”, one study simply mentioned “potentially inappropriate medications” without further explanation [79] and the other study included examples of potentially inappropriate medications from existing literature such as duplication of medications, drug-drug interactions, medications used to treat side effects of other medications and medications which are unnecessary for a specific patient [10]. Only one study explicitly defined appropriate polypharmacy, which was defined as “the optimisation of medications for patients with complex and/or multiple conditions where medicine usage agrees with best evidence” [117].

Four studies (3.6%) used polypharmacy tools or criteria to identify potentially inappropriate medications [10, 114, 116, 118]. The Beers criteria as an indicator of potentially inappropriate medications were used in all four (three studies used Beers criteria 2003 and one used Beers criteria 1997) [10, 114, 116, 118]. One study used the Medication Appropriateness Index (MAI) and the Healthcare Effectiveness Data and Information Set (HEDIS) [114]. None of the studies explicitly identified the need to distinguish between appropriate and inappropriate polypharmacy based on the pharmacology of medications involved, how they interact with each other and comorbidities for a specific patient.

Of the 110 studies included in the review, only one highlighted the inconsistencies in the definitions of polypharmacy in the literature. The authors of this study suggested that polypharmacy be defined as patients visiting multiple pharmacies which may be associated with safety concerns relating to potential outcomes such as medication duplication, drug-drug interactions and adverse effects [112].

## Discussion

The results of this systematic review show that there is large heterogeneity in the definition of polypharmacy; ranging from numerical counts only, numerical counts for a given duration of therapy or setting or descriptive, which included terms such as minor, moderate, major and excessive polypharmacy. The lack of a clear and universal definition of polypharmacy as well as terms such as minor, moderate major polypharmacy makes it challenging for healthcare professionals to assess and consider efficacy and safety issues within the clinical setting.

The most commonly reported category of definitions for polypharmacy and associated terms was numerical only. The most commonly used term was polypharmacy which was defined as five or more medications by 46.4% of studies (51 articles). There was a wide range of numerical only definitions of polypharmacy, ranging from two or more medications to 11 or more medications. However, the clinical basis for using a numerical count such as five or more medications to define polypharmacy and the potential of this to rationalise medication use and optimise health outcomes is not elucidated in most studies. It has been postulated that while the term polypharmacy has evolved over time, the basis for the definition is simply more drugs being prescribed or taken than are clinically appropriate in the context of a patient’s comorbidities [12]. It is commonly reported that as the number of prescribed drugs increases, so do the chances of adverse drug events and likelihood of harm [120]. However, the specific number of drugs taken is not itself indicative of appropriateness of therapy as all of the drugs may be clinically necessary and appropriate for the patient. Despite this, only one study argued that instead of using numerical counts of medications, clinicians should identify appropriateness of therapy, where potential benefits outweigh the potential harms [121]. There is a clear need towards adopting the term ‘appropriate polypharmacy’ in order to differentiate between the prescribing of ‘many’ and ‘too many’ drugs instead of a simple numerical count of medications, which is of limited value in practice [11, 12, 120–122].

Whilst the addition of duration of therapy or healthcare setting to a count of medicines in the definition of polypharmacy provided more specific definitions, it did not provide any further clarity or consistency. Five or more medications were again used in the definitions but with a period of time attached such as 90 days or more [104]. The use of duration in the definitions appeared to be included to identify those patients with longer term or chronic use of medications, potentially identifying those patients who might be at greatest risk of medication-related problems. Definitions incorporating a healthcare setting commonly used five or more medications as the count, but using a setting such as at the time of hospital discharge [106].These definitions were largely based on the dispensing data available to assess prevalence and incidence of polypharmacy, rather than an evidence based approach of determining appropriateness of therapy; the setting provided little addition to existing definitions of polypharmacy in a clinical sense of medication rationalisation and minimisation of harm.

A recent commentary on polypharmacy stated that while a large body of literature confirms the fact that patients are increasingly taking large numbers of medications, numerical definitions of polypharmacy do not ascertain the clinical appropriateness of therapy and the process of rationalising those medications [123]. The author argued that when each of the medications can be linked to practice guidelines for chronic conditions for a given patient, using a numerical cut-off to define polypharmacy becomes irrelevant. While the number of medications can be a starting point, medications should be assessed in terms of their indication, efficacy and potential for harm with each other (not in isolation) given pharmacokinetic and pharmacodynamics interactions, in order to facilitate deprescribing of inappropriate medications [123, 124]. Medications should be assessed for risks and benefits and the final combination of medications should be based on benefits outweighing the risks [124, 125]. Current literature is alluding to looking beyond single disease management guidelines and considering the patient’s complete scenario by considering all comorbidities and medications being prescribed for a given patient to consider the patient as a whole and focusing on improving the overall health [11, 12, 117, 120–122, 125].

While the use of multiple medicines may be clinically appropriate for some patients, it is important to identify those patients who may be at risk of adverse health outcomes as a result of inappropriate polypharmacy. Only seven studies recognised the distinction between appropriate and inappropriate polypharmacy. This is crucial to facilitate the deprescribing of inappropriate medications and optimal use of appropriate medications. Consideration of comorbid conditions and other medications is required to make definitions clinically relevant, to facilitate medication assessment and rationalisation in every day practice. While a small number of studies (3.6%) used polypharmacy tools or criteria including the Beers criteria, MAI and HEDIS to identify potentially inappropriate medications to detect potentially inappropriate medications, the limitations of the tools and criteria in the everyday clinical setting were recognised [10, 114, 116, 118].The Beers criteria which was used in all four studies identified, is a commonly used prescribing assessment tool based on a list of potentially inappropriate medications to be avoided in the older population [126, 127]. However, it has recognised limitations such as requiring regular updates to ensure clinical relevance and including a list of outdated medications which may not be used in practice at that time.

### Strengths and limitations

Strengths of this systematic review include the novelty of summarising the range of polypharmacy definitions available in literature. A comprehensive search strategy of three large and reliable databases (MEDLINE, EMBASE and Cochrane) was used, meaning that it is likely that all relevant articles were identified.

A limitation of this review is the inclusion of studies in English only which can cause information bias. While EMABSE, MEDLINE (Ovid) and Cochrane databases were searched, the absence of other databases such as Scopus could have introduced selection bias. Additionally articles from the year 2000 until present have been included. There may be clinically relevant definitions for polypharmacy which were added to literature prior to 2000 which have not been included in this review. While authors discussed the inclusion criteria and data being extracted, there is still the potential for confusion bias.

## Conclusions

While the most commonly used definition of polypharmacy is being on five or more medicines, definitions are variable, which can cause confusion for researchers as well as clinicians in practice. Numerical definitions of polypharmacy do not account for specific comorbidities present and make it difficult to assess safety and appropriateness of therapy in the clinical setting. There is a need for an internationally agreed definition of polypharmacy. The results indicate the need for a shift towards the term ‘appropriate polypharmacy’ using a holistic approach of assessing medication use in context of comorbidities present, according to best available evidence in order to optimise health outcomes.

## Abbreviations

HEDIS: Healthcare Effectiveness Data and Information Set
MAI: Medication Appropriateness Index
PRISMA: Preferred Reporting Items for Systematic reviews and Meta-Analyses
